# Ameliorative effect of taurine against diabetes and renal-associated disorders (Review)

**DOI:** 10.3892/mi.2021.3

**Published:** 2021-05-31

**Authors:** Stella Baliou, Maria Adamaki, Petros Ioannou, Aglaia Pappa, Mihalis I. Panayiotidis, Ioannis Christodoulou, Demetrios A. Spandidos, Anthony M. Kyriakopoulos, Vassilis Zoumpourlis

**Affiliations:** 1 Institute of Chemical Biology, National Hellenic Research Foundation, 11635 Athens, Greece; 2 Department of Internal Medicine and Infectious Diseases, University Hospital of Heraklion, 71110 Heraklion, Greece; 3 Department of Molecular Biology and Genetics, Faculty of Health Sciences, Democritus University of Thrace, 68100 Alexandroupolis, Greece; 4 Department of Cancer Genetics, Therapeutics and Ultrastructural Pathology, The Cyprus Institute of Neurology and Genetics, 2371 Nicosia, Cyprus; 5 Cyprus School of Molecular Medicine, 2371 Nicosia, Cyprus; 6 Laboratory of Clinical Virology, Medical School, University of Crete, 71409 Heraklion, Greece; 7 Nasco AD Biotechnology Laboratory, 18536 Piraeus, Greece

**Keywords:** taurine, diabetes, diabetic nephropathy, renal transplantation

## Abstract

To develop novel therapeutic methods for both diabetic and renal disorders, scientists had initially focused on elucidating the molecular mechanisms of taurine in established cell lines and mouse models. Although a large amount of data have been revealed, taurine has been confirmed to be the next step of novel promising therapeutic interventions against diabetic disorders. Taurine appears to ameliorate diabetes 1-related complications in various organs through its antioxidant, anti-inflammatory and anti-hormonal actions. In type 2 diabetes, taurine has been positively implicated in glucose homeostasis, exerting potent hypoglycemic, anti-obesity, hypotensive and hypolipidemic effects. Of particular interest is that taurine provides protection against renal dysfunction, including hypertension and proteinuria, specific glomerular and tubular disorders, acute and chronic renal conditions, and diabetic nephropathy. The ameliorative effects of taurine against renal disorders are based on its osmoregulatory properties, its association with signaling pathways and its association with the renin-angiotensin-aldosterone system (RAAS). Further clinical studies are required to ensure the importance of research findings.

## 1. Types of diabetes

Diabetes is a prevalent endocrine disease associated with oxidative stress. In 2014, 422 million individuals were diagnosed with diabetes worldwide, while diabetes was directly associated with 1,5 million deaths in 2012 and 2,2 million deaths indirectly through an increased risk of cardiovascular mortality and other diseases (1).

Diabetes mellitus is categorized into two types according to insulin dependence. Type 1 diabetes mellitus or insulin-dependent diabetes mellitus (IDDM) (formerly known as juvenile diabetes) is characterized by hyperglycemia and hypoinsulinemia. Type 1 diabetes mellitus is considered an autoimmune disease, in which T-cells mediate the elimination of pancreatic β-cells and thereby contribute to the production of low insulin levels (2). In type 2 diabetes mellitus or non-insulin dependent diabetes mellitus (NIDDM) (formerly known as adult diabetes), insulin resistance seems to be the predominant factor and occurs from defects in insulin secretion and a low tissue sensitivity to insulin (3). Diabetes is also known to cause complications, such as cardiovascular diseases, neuropathy, nephropathy, retinopathy, foot ulcers, skin lesions and hearing impairment (4).

Diabetes mellitus is associated with high blood sugar levels for a long period of time due to alterations in carbohydrate, protein and fat metabolism, which results from a dysfunction in insulin secretion, insulin action, or both (1). In diabetic conditions, excessive reactive oxygen species (ROS) formation mainly appears to be responsible for pancreatic β-cell dysfunction and insulin resistance (5). When ROS are produced by the mitochondria, they cause an impairment in the mitochondrial respiration chain activity, which may, in turn, lead to the excessive formation of superoxide anions (O_2_^-^), and thus contributing to the incidence and pathogenesis of diabetes (6). The underlying mechanisms of ROS through the production of superoxide anions contribute to the pathogenesis of diabetes by upregulating poly(ADPribose) polymerase (PARP) and suppressing the action of glyceraldehyde-3 phosphate dehydrogenase (GAPDH), which constitutes an important glycolytic enzyme (7). Subsequently, hyperglycemia-induced superoxide anions and hyperglycemia induce the flux of mitochondrial electron transport chain through four damaging pathways [generation of advanced glycation end-products (AGEs), protein kinase C (PKC) activation, polyol formation and hexosamine pathway stimulation], thus supporting the hypothesis that mitochondrial-derived ROS is the missing link to the glucose disturbance observed in diabetes (7). Various mechanisms have also been proposed to enhance the oxidative stress mediated by diabetes, such as lipid peroxidation (LPO), decreased antioxidant activity and reduced glutathione (GSH) levels (8). For example, cerebral cells isolated from streptozotocin (STZ)-treated rats are characterized by increased levels of malondialdehyde (MDA), increased LPO and a concomitant reduction in antioxidant enzyme activity and in the glutathione-to-glutathione disulfide (GSH/GSSG) ratio (9). In addition to the above, other mechanisms such as glucose auto-oxidation and protein glycation can be important factors in determining the incidence of diabetic complications (10).

Normal metabolism and energy production rely on the multiple actions of taurine (11). The antioxidant properties of taurine have been demonstrated in a wide range of distinct diabetic animal models, where it was shown to provide protection from the stressful signals of insulin resistance or obesity, via various mechanisms: i) The upregulation of antioxidant enzymes, such as superoxide dismutase (SOD), catalase (CAT) and glutathione peroxidase (GPx); ii) interference with PKC activity; iii) the downregulation of nicotinamide adenine dinucleotide phosphate (NADPH) oxidase/cytochrome P450 2E1 (CYP2E1) expression ratio; iv) the inhibition of protein carbonylated (PC) content accumulation; v) the inhibition of LPO; vi) the disruption of the generation of AGEs (12-15). Based on the above, taurine could be used as an effective therapeutic agent against diabetic complications, mostly due to its anti-oxidant activity.

## 2. Beneficial effects of taurine on type 1 diabetes

Historically, the first line of evidence that taurine exerted a positive effect on glucose tolerance in diabetic patients, through the activation of glycolysis and glycogenesis, was provided in 1976(12). Following this, an inverse association seemed to exist between taurine and plasma glucose content in patients with type 1 diabetes (13).

Type 1 diabetes is a condition that can be easily replicated in animal models using either streptozotocin (STZ) or alloxan toxins, which impair the function of pancreatic β-cells (14,15). The underlying mechanisms of toxins is based on the induction of alkylated DNA damage and cell death (16), contributing to increased PARP levels and to a decreased adenosine triphosphate (ATP) content (15,17). The STZ or alloxan-mediated diabetic animals present with lower plasma taurine levels than normal animals (18). In this context, the livers of diabetic animals are characterized by markedly reduced concentrations of taurine, which may be due to an impairment in taurine transporter (TauT) activity under high glucose conditions and to an intracellular accumulation of sorbitol sufficient to abrogate intracellular levels of taurine (19). As anticipated, taurine emerged as a promising therapeutic agent for STZ-treated rats (18), alloxan-treated rats (20,21) and alloxan-treated rabbits (22), in terms of eliciting a hypoglycemic effect.

### Antioxidant effects of taurine against type 1 diabetes

The beneficial effects of taurine on hyperglycemia caused by diabetes, have been validated by a number of studies. For example, taurine has been shown to provide protection against the biochemical, functional and morphological changes caused by diabetes in the plasma, erythrocytes (23) and kidneys of rats previously treated with STZ (24). In another example, plasma glycated hemoglobin (HbA1c), cholesterol/triglyceride levels and plasma LPO products were reduced in STZ-treated diabetic rats following treatment with taurine prior to the diabetic onset (25). Similarly, the inhibitory effect of taurine on glucose levels seems to be achieved by suppressing hyperglycemia in the type 1 model of diabetes induced by alloxan, highlighting a potential inhibitory effect of taurine against hyperglycemia prior to the diabetic onset (22). Notably, the potential of taurine on altering the hyperglycemic status became evident in various models of type 1 diabetes, thereby affording protection against diabetic complications induced by STZ or alloxan, independently of the diabetic onset (21). In some cases, the hypoglycemic effect of taurine was evidenced when administered at the time point of the diabetic onset (26), whereas in other cases, its effect was manifested after the STZ-induced diabetic onset (27). In both cases, it was demonstrated that taurine ameliorated the stress signals caused by hyperglycemia and was thus beneficial in controlling glucose homeostasis (26,27). In other words, as taurine was able to reverse the phenotype of aorta rings derived from STZ-treated diabetic rats, causing a reduction in the response to norepinephrine and an increase in the response to acetylcholine (28), it was concluded that it improves the impaired endothelium-dependent vasodilator response in hyperglycemia (28).

### Ameliorative effects of taurine against endothelial dysfunction and lipoprotein accumulation in type 1 diabetes

**Figure 1:**
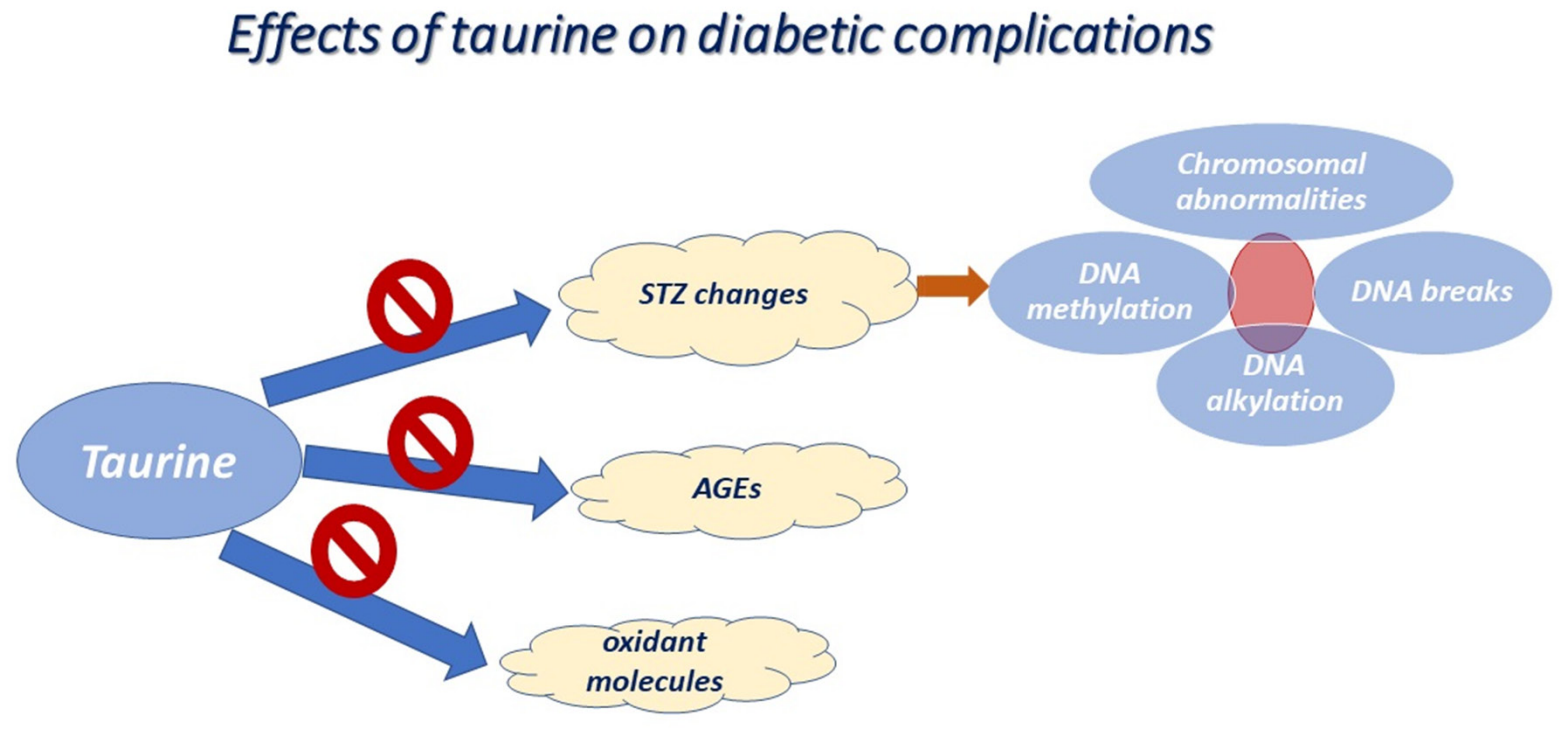
Protective effects of taurine on type 1 diabetes-induced complications. Taurine confers protection against diabetes through reduction of signals mediated by hyperglycemia and oxidative stress. STZ, streptozotocin; AGEs, advanced glycation end-products.

When taurine is administered for long periods of time, it appears to confer protection against endothelial dysfunctional cell-mediated signals, by reducing the levels of AGEs and of various oxidant molecules, including oxidized low-density lipoprotein (ox-LDL) and hypochlorous acid (HOCl) (summarized in Fig. 1) (29,30). It also appears to ameliorate endothelial dysfunction, leading to the formation of low quantities of MDA (31). Consistent with the above, taurine plays an essential role in maintaining the extracellular matrix (ECM) and the junctions between endothelial cells. When endothelial cells are cultured under high glucose conditions, the upregulation of adhesion molecules, including vascular cell adhesion molecule-1 (VCAM-1) and intercellular adhesion molecule-1 (ICAM-1), is observed (29). *In vivo*, taurine supplementation for 5 days appears to be sufficient in altering leukocyte-endothelial cell interactions and hyperglycemia-induced endothelial apoptosis (32). In the same frame, the cytoprotective role of taurine against type 1 diabetes emerged through its effect on cholesterol levels. It has been demonstrated that taurine supplementation causes the inhibition of lectin-like ox-LDL receptor-1 (LOX-1), which is located in the endothelial cells of aortas, in STZ-treated diabetic rats (33). In addition, the chronic administration of taurine has been proven to be very helpful in inhibiting the increase in LDL cholesterol levels in STZ-treated diabetic mice (34). Accordingly, taurine has been shown to attenuate the incidence of the atherosclerosis-associated decrease in high-density lipoprotein (HDL) cholesterol levels in plasma (34), which occurs when lipid-forming fatty acids adhere to the walls of arteries in patients with atherosclerosis.

### Ameliorative effects of taurine against oxidative stress in hepatocytes in type 1 diabetes

**Figure 2:**
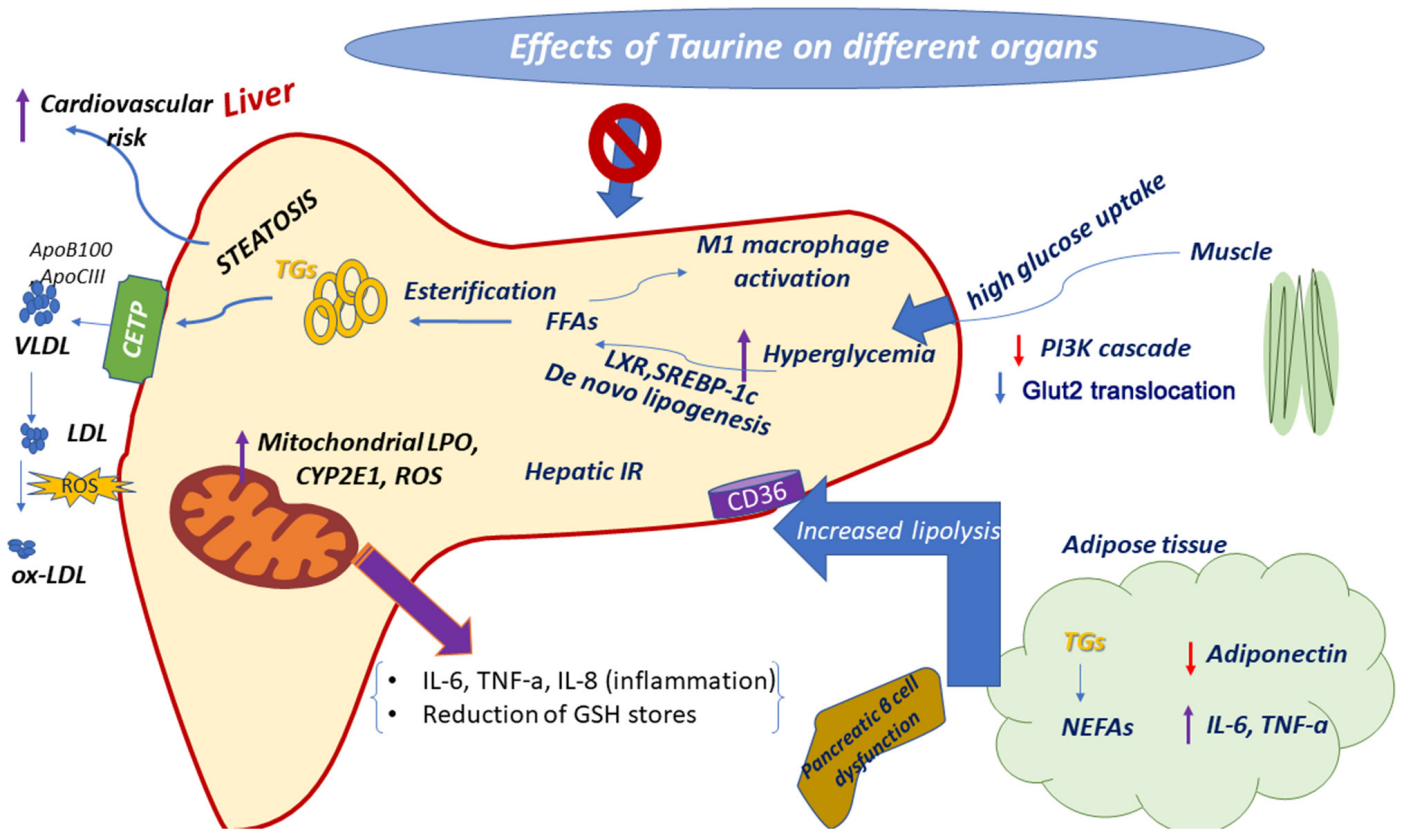
Therapeutic properties of taurine against hyperglycemia. In the diabetic liver, hepatic glucose uptake is increased and the blood glucose level, as well liver disease are induced. Taurine protects against hyperglycemia via inducing distinct pathways. Purple arrows indicate upregulation and red arrows indicate downregulation. TNF-α, tumor necrosis factor-α; IL, interleukin; CYP2E1, cytochrome P450 2E1; LPO, lipid peroxidation; ROS, reactive oxygen species; FFAs, free fatty acids; SREBP-1c, sterol regulatory element-binding protein 1c; LXR, liver X receptor; PI3K, phosphoinositide 3-kinase; GLUT2, glucose transporter 2; NEFAs, non-esterified fatty acids; TGs, triglycerides; CETP, cholesteryl-ester transfer protein; LDL, low-density lipoprotein; VLDL, very-low-density lipoprotein; ox-LDL, oxidized low-density lipoprotein.

In hepatocytes of animal models with type 1 diabetes, the beneficial role of taurine has been highlighted through its antioxidant effects. Initially, Mohamed and Gawad (35) observed that taurine reduces diabetes-mediated oxidative stress, thereby promoting the survival of hepatocytes from STZ-induced harmful stimuli in rats. There are numerous mechanisms through which taurine attenuates diabetes-associated hepatic stress, as summarized in Fig. 2. Firstly, taurine appears to reverse oxidative stress-related hepatic injury by reducing CYP2E1 activity and gene expression (18). This makes sense if one considers that CYP2E1 is a form of cytochrome P450, which is involved in the catabolism of endogenous compounds and in free radical reactions (36), thereby highlighting the potential redox orchestration by taurine. In the same frame, Fukuda *et al* (37) supported the antioxidant activity of taurine, by demonstrating its ability to eliminate LPO products in an indirect manner; taurine appeared to be involved in increasing hepatic fatty acid oxidation and therefore hepatic efflux of hepatic fatty acids to renal cells. Similarly, the beneficial effect of taurine on the hepatic disturbance was also supported by the activation of hepatic phosphoinositide 3-kinase (PI3K), protein kinase B (Akt) and hexokinase, which led to the reduced translocation of glucose transporter (GLUT)2 to the membrane of hepatocytes in alloxan-induced diabetic rats, highlighting the capacity to interfere with multiple signaling pathways (38).

### Ameliorative effects of taurine on GSH levels in type 1 diabetes

Taurine exerts its beneficial effect on type 1 diabetes through alterations in the cellular GSH content. As previously demonstrated, following treatment with taurine, the GSH content and the GSH/GSSG ratio appeared to be increased by 14 and 27%, respectively. In this manner, a protective mode of action by taurine against STZ-induced oxidative stress in the brain and spinal cord areas of diabetic rats was suggested (39). In another example, taurine was proven to restore pancreatic β-cell damage when administered at 1.2 mM per kg in male Sprague-Dawley rats within 45-75 min prior to the intraperitoneal injection of STZ in rats (39). In the same frame, it has been proposed that taurine protects against oxidative stress by serving as an inducing signaling cue for GSH-related enzymes, such as glutathione reductase (GR) and glutathione synthetase (GSS), thereby contributing to the maintenance of the intracellular GSH stores in the liver (40). Taurine has been shown to improve hepatic GPx activity and to increase GSH levels by directing cysteine into the GSH synthesis pathway in the liver, proving its antioxidant properties in the liver (18). Accordingly, Furfaro *et al* (41) proved that the administration of taurine to STZ-treated rats for 6 months was sufficient to inhibit the loss of the hepatic GSH content and the decrease in the GSH/GSSG ratio. In a similar manner, in another study, 1% taurine supplementation in drinking water was shown to exert an antioxidant and hypoglycemic effect in alloxan diabetic rabbits, by increasing the GSH/GSSG ratio and by restoring intracellular GSH levels through increasing renal GR activity and by inhibiting hydroxyl radicals, as well as by increasing the activity of antioxidant enzymes, such as CAT in the serum and renal cortex (22). Taurine has also been shown to exhibit neuroprotective and antioxidant activity by reducing LPO and increasing GSH levels, thereby providing protection to rat cerebral cells subjected to injury from D-galactose-related stress (42).

### Positive effects of taurine on insulin secretion in type 1 diabetes

Τhe inhibitory action of taurine on insulin-dependent diabetes has been established in the pancreas, through experiments proving the significant contribution of taurine, conferring protection to pancreatic β-cells against injury and the preservation of normal secretory granule functions, thus delaying the onset of diabetes (43). In an experimental set-up where taurine was administered to diabetic rats at the onset of diabetes, in which STZ (60 mg/kg i.p.) was administered for 14 days, there was a marked decline in plasma/blood glucose levels within 6 weeks of diet (44). Furthermore, pancreatic islets isolated from rats fed a high-glucose low-protein diet, which were characterized by markedly reduced insulin secretion rates, began to produce insulin again following the taurine administration (45). Similarly, in another study, taurine supplementation seemed to play an important role in promoting insulin secretion in the islets of malnourished mice fed a high-fat diet (HFD) (46). The long-term administration of taurine was sufficient to restore the function of impaired pancreatic islet cells, by inducing insulin secretion. At the molecular level, Lin *et al* (47) revealed that the transcriptional regulation of the insulin response in islet cells from rats with STZ-induced diabetes occurred *in vitro* following the taurine administration. In particular, taurine promoted insulin secretion by enhancing the transactivation of transcription factors, such as pancreatic duodenal homeobox- 1 (Pdx-1) and neurogenic differentiation 1 (NeuroD1) (47). Consistent with the results obtained *in vitro*, in another study, pancreatic islet cells isolated from taurine-treated diabetic mice were characterized by markedly high Pdx-1 levels of transcription within 30 days of treatment, resulting in increased glucose-activated insulin secretion (48). Notably, another study also demonstrated that the mRNA levels of MafA, neurogenin 3 (Ngn3) and NeuroD1 transcription factors also appeared to increase in mice following the administration of taurine (49). Those findings were important considering that Pdx-1, NeuroD1 and MafA are the crucial transcription factors that bind to the upstream regions of the insulin gene promoter, thereby determining the rate of insulin synthesis (50), with NeuroD1 being essential for the survival and normal functioning of pancreatic cells (51). Similar results were obtained in diabetic mice which had 2% taurine supplemented in their drinking water for 30 days (48); the animals presented with low blood glucose levels and supplementation of taurine appeared to increase both the basal and the insulin-activated tyrosine phosphorylation of the insulin receptors in the skeletal muscle and liver of diabetic mice (48). The mice in both groups (taurine versus no taurine) exhibited glucose-induced insulin release; however, the mice in the taurine group exhibited higher levels of insulin secretion compared with the control group. The expression levels of the genes involved in the activation of insulin secretion, i.e., GLUT2, glucokinase, sulfonylurea receptor-1 (SUR-1) and pancreatic duodenal homeobox-1 (Pdx-1) transcription factor were increased in the taurine group (48). In another example, it was demonstrated that the protective mode of taurine against diabetic-associated pancreatic dysfunction relied on its anti-inflammatory properties. In particular, pregnant non-obese diabetic (NOD) mice were administered taurine until weaning, in order to investigate the effect of taurine on pancreatic alterations in autoimmune type 1 of diabetes (43). The results suggested that taurine modulates the infiltration of mononuclear leucocytes into the pancreatic islets, thereby reducing the incidence of diabetes by 20% (43).

### Neuroprotective effects of taurine through the activation of inhibitory neurotransmitters

If one considers that cerebral cells are more susceptible to oxidative damage than other types of cells mainly due to their strict oxygen requirements, peroxidative damage to lipids and proteins, and the lack of antioxidant responses, it is thus expected that diabetes will have multiple effects on the brain (52). In the long term, diabetes exerts detrimental effects on cerebral cells, such as altered redox potential, tissue damage, accelerated cognitive impairment, brain atrophy and brain aging (9). Above all, an imbalance arises between the overproduction of free radicals/nitrogen species, and the diminished activity of anti-oxidant enzymes, leading to an altered redox metabolism, mitochondrial dysfunction and compromised energy metabolism (53). In this context, taurine exhibits potent neuroprotective activity and is linked to a lower risk of diabetic neuropathy. In a previous study, in the hippocampus of STZ-treated diabetic mice, taurine increased the transcriptional levels of the GABAA receptor (GABAAR) α2 subunit and of the brain-derived neurotrophic factor (BDNF), thereby leading to an increased synthesis of gamma-aminobutyric acid (GABA) by glutamate decarboxylases (GAD65 and GAD67) (54). In other words, taurine has been shown to act as a GABAAR agonist in synaptic and extrasynaptic membranes, by activating the neurotransmitter system and counterbalancing the lower extracellular levels of GABA in STZ-treated mice (55). Chronic treatment with taurine has been shown to increase the expression levels of BDNF, which appear to be very low in the hippocampus of STZ-treated rats, thereby rescuing neurons from atrophy (56). Notably, this effect appears to co-exist with alterations in nerve conductance deficits, hyperalgesia and nerve blood flow (57). For example, taurine has been shown to attenuate the defects of hind limb sciatic motor and digital sensory nerve conduction velocity, nerve blood flow and sensory thresholds in Zucker diabetic fatty rats (58). Moreover, taurine seems to improve excessive sympathetic nervous system activity and diuretic action through the activation of sodium secretion and maintenance of potassium and magnesium (59). The administration of taurine also targets ROS formation, causing a remarkable decline in intracellular calcium levels in the mitochondria of neurons (60). Taking all of the above-described evidence into account, it can be suggested that taurine has the potential to bypass diabetic neuropathy, by mainly activating nerve growth factors as weapons against excessive oxidative stress.

### Neuroprotective effects of taurine through the attenuation of oxidative stress, inflammation and the hormonal axis

Taurine exerts potent anti-inflammatory and antioxidant activity through which it weakens neural responses in the diabetic context (61). Taurine reduces the expression levels of nuclear factor κ light chain- enhancer of activated B cells (NF-κB) and increases the expression levels of nuclear factor erythroid-derived 2-like 2 (Nrf2), of heme oxygenase (HO-1) and GLUT1 and 3 in the brains of diabetic rats, as compared to healthy rats (61). The administration of taurine seems to partially lower the serum MDA content and neuroinflammation via the inhibition of NF-κB expression, and an increase in Nrf2, HO-1 and GLUT1/3 expression levels in diabetic rats (61).

The regulatory effects of taurine on diabetes have not only been linked to its antioxidant properties, but also to potent hormonal alterations caused by the inhibition of the hypothalamic-pituitary-gonadal axis in male diabetic rats (35). Specifically, it has been proven that the plasma levels of acetylcholinesterase (AChE), gonadotropic, gonadotropin-releasing hormone (GnRH), thyroid-stimulating hormone (TSH), follicle-stimulating hormone (FSH) and luteinizing hormone (LH) are reduced in diabetic animal models through the exogenous administration of taurine (62). These changes are accompanied by a marked elevation in the levels of thyrotropin-releasing hormone (TRH), T3 and thyroxin (35). In support of this, it has been shown that taurine decreases hyperglycemia, insulin loss and mitochondrial oxidative stress, as well as hormone-associated changes through the inhibition of the hypothalamic-pituitary-gonadal axis (62).

## 3. Effects of taurine on type 2 diabetes

Type 2 diabetes is a major contributing factor for the development of cardiovascular diseases, which are the first cause of mortality worldwide. To address this challenge, experimental animals on high-fat or high-sucrose diets have become models of both liver and skeletal muscle insulin resistance (63). Nonetheless, there is some controversy regarding mitochondrial function in type 2 diabetes, with the majority of studies pointing towards a reduction in the oxidative potential of mitochondria (64) and other studies pointing towards an increased oxidative potential (65,66).

Several studies have proposed that disturbed taurine homeostasis accounts for the high incidence in obesity and diabetic complications. It has been shown that the taurine concentration is low in the plasma and platelets of patients with type 2 diabetes (67), as well as in the plasma of diabetic animal models (67). In this context, a lower dietary intake of taurine is associated with a higher cardiovascular risk (68). This suggests that diabetes may be a taurine-deficient condition, as supported by the low intestinal absorption rates and the high renal excretion rates of taurine in these patients (69).

If one considers that taurine is the key element of mitochondrial oxidative phosphorylation, it can be suggested that taurine acts protectively against diabetes mellitus, insulin resistance and related complications (70). The cytoprotective effect of taurine has been demonstrated in different cells of type 2 diabetic animals, through various mechanisms. For example, taurine supplementation has been shown to improve hyperglycemia and insulin resistance in Otsuka Long-Evans Tokushima Fatty (OLETF) rats and to reduce diabetic complications, including retinopathy, nephropathy, neuropathy and cardiomyopathy (19,71). However, clinical trials have been conducted in order to investigate the hypoglycemic effects of taurine on type 2 diabetes, although no such properties have been reported thus far (72).

The protective mode of taurine against non-insulin-dependent diabetes mellitus, and insulin resistance, has been reported via multiple mechanisms (21,73,74). This is possible as taurine is involved in a number of important physiological processes; for example, taurine is positively implicated in glucose homeostasis, exerting a strong hypoglycemic effect (20), by reducing oxidative stress (75) and inflammation (76,77), and by increasing insulin sensitivity and insulin secretion (78). Another mode of action through which taurine improves diabetic complications is the reduction of mitochondrial calcium overload, usually accompanied by appropriate protein folding (79). For example, taurine has been shown to reduce hyperalgesia and abnormal calcium signaling in the sensory neurons of diabetic rats (57).

### Advantageous effects of taurine on diabetes 2 through its antioxidant properties

Taurine has been revealed to exert its anti-diabetic effects by rescuing pancreatic β-cell dysfunction through its antioxidant capacity (80). As regards the antioxidant properties of taurine, it has been reported that taurine ensures normal electron transport chain (ETC), thereby protecting the mitochondria from excessive O_2_^-^ formation (75). The loss of taurine in the mitochondria causes a significant reduction in the biosynthesis of mitochondrial-encoded proteins ND5 and ND6, resulting in the disruption of complex I and III activities of the respiratory chain (81). This is probably due to the inadequate incorporation of taurine into mitochondrial tRNA (82,83). In the same context, taurine exerts its anti-oxidant action via its capacity to counteract changes caused by overproduction of uncoupling protein 2 (UCP2) in pancreatic β-cells, thereby neutralizing the possibility for excessive mitochondrial O_2_^-^ generation (84). Taurine has also shown to abrogate LPO products in genetically hyperlipidemic animals, including apoE-deficient mice (85) and Watanabe heritable hyperlipidemic (WHHL) rabbits, that mimick human familial hypercholesterolemia (86). In obese malnourished mice, taurine maintains whole-body glucose tolerance and promotes liver insulin signal transduction, as shown by the closely linked regulatory importance of taurine to redox balance and protein phosphatases activity (87). Taurine has also been shown to mediate its antioxidant properties in muscles isolated from high-glucose fed mice within 6 h of treatment (88).

### Beneficial effects of taurine on diabetes 2 through its anti-inflammatory properties

Inflammation has been considered a major contributing factor to the pathogenesis of diabetes; thus, it is plausible that macrophages infiltrate into the adipose tissue of patients with type 2 diabetes (89). It is common knowledge that taurine can be converted into taurine chloramine, which exerts anti-inflammatory activity by preventing the nuclear translocation of NF-κB transcription factor and consequently by inhibiting the expression of pro-inflammatory cytokines [tumor necrosis factor-α (TNF-α) and monocyte chemoattractant protein 1 (MCP-1)] (76,77). For example, taurine has been shown to alleviate hyperglycemia symptoms in C57BL/6 mice on a HFD, through its anti-inflammatory action. In particular, taurine has been reported to promote the M1 to M2 conversion of macrophages in the murine adipose tissue and to inhibit the gene expression of pro-inflammatory cytokines (90). The short-term administration of taurine appears to be crucial for improving glucose homeostasis through its anti-inflammatory action, by reducing the expression levels of pro-inflammatory c-Jun NH-terminal kinase 1 (JNK1) in the liver of rats on a HFD (91). Furthermore, You *et al* (90) demonstrated that rats with HFD-induced obesity presented a recession of diabetic signs following 8 weeks of 3% taurine supplementation in their drinking water, possibly due to the positive anti-inflammatory action of taurine on adiponectin and cholesterol levels. In the same context, other researchers have also supported that taurine improves insulin sensitivity and LPO through enhancement of adiponectin levels in obese women (92).

### Beneficial effects of taurine on diabetes 2 through its hypoglycemic properties

Taurine exerts its hypoglycemic properties through its ability to improve peripheral insulin sensitivity, and in this manner, by enhancing insulin release in response to glucose uptake (78). Following treatment with taurine, insulin secretion is induced, by upregulating insulin receptor substrate 1 (IRS-1/2) tyrosine and Akt serine phosphorylation in diabetic conditions (91). Experiments with malnourished mice on a HFD have shown that central insulin signaling is a determinant of altered food intake (78). Specifically, as previously demonstrated, mice which had already been on a balanced diet or on a protein-restricted diet for 6 weeks, were fed a HFD for another 8 weeks (78). Taurine supplementation (5%) appeared to reverse the features of obesity only in mice fed a normal diet before being fed a HFD (78). A decrease in hyperglycemia was only observed in taurine-treated mice previously fed a balanced diet, possibly due to taurine reducing the activated form of insulin receptor [phosphorylated IRS-1 (p-IRS-1)] by half, without altering its total protein levels in the murine hypothalamus (78). In other words, taurine seemed to promote insulin secretion and to restore metabolic disturbances in experimental animals fed a balanced diet before being fed a HFD, possibly due to the accumulation of protein constituents which are sufficient to compensate for the metabolic alterations caused by a HFD (78). In another case, taurine supplementation appeared to improve insulin sensitivity by restoring the phosphorylation status of IRS and Akt in rats subjected to lipid infusion-induced insulin resistance; the underlying mechanism was considered to have occurred via inhibition of the inflammatory JNK1 in the liver of rats (91). The insulin-like properties of taurine also seem to be mediated by a reduction of ATP-sensitive K^+^ channels, which are crucial for insulin secretion (93,94). Other possible mechanisms for the taurine-mediated increase in insulin sensitivity include increased glycogen synthesis in the liver and glucose uptake in the peripheral tissues, both of which imply an indirect association between taurine and insulin sensitivity (88).

Furthermore, the high potency of taurine in preventing diabetic complications is considered to stem from its ability to significantly decrease glucagon, thereby contributing to the maintenance of glucose homeostasis (49). Numerous studies have described the beneficial hypoglycemic effect of taurine on increasing insulin availability, through the activation of hepatic glucose accumulation as glycogen (21) and the inhibition of gluconeogenesis in animal models of diabetes (95). The positive effect of taurine on glucose regulation has also been confirmed via a more prominent formation of islet-like cell aggregates (ICAs) from autologous adipose-derived mesenchymal stem cells (ADMSCs), following the administration of taurine (96).

### Beneficial effect of taurine on diabetes 2 through its interference with energy expenditure

The anti-obesity effects of taurine have been well-documented in genetically modified diabetic KK mice (97), OLETF rats (71) and mice on a HFD (98). The observation that taurine inhibits postprandial glucose oxidation suggests that it may be responsible for altering the proportion of energy expenditure in glycogen synthesis or lipogenesis (29). The main underlying mechanism is the increase in oxygen consumption rates (98). Specifically, the dietary supplementation of taurine has been shown to increase the expression levels of energy expenditure-related genes, such as peroxisome proliferator-activated receptor α (PPARα), peroxisome proliferator-activated receptor gamma co-activator 1 (PGC-1α) and Nrf2, and their target genes [lipoprotein lipase (LPL), acyl-CoA oxidase (ACO), acyl-CoA synthetase (ACS) and medium-chain acyl-CoA dehydrogenase (MCAD)] in adipose tissue (98). If one considers the already established cholesterol and lipid-lowering properties of taurine, it is only reasonable to suggest that taurine is also actively involved in increasing the rate of lipolysis in adipocytes, thereby acting against obesity. This property may be due to either direct activation of cyclic adenosine monophosphate (cAMP)-dependent protein kinase A (PKA) catalytic activity or to an indirect PKA stimulation through the formation of cAMP and interference with hydrogen peroxide (H_2_O_2_) accumulation (99). For example, 2.5% taurine supplementation in the drinking water of rats with mono-sodium glutamate (MSG)-induced obesity for 10 weeks is sufficient to attenuate lipid accumulation, inhibiting total fat and triglyceride formation, without altering glucose homeostasis (100). In rats with MSG-induced obesity, taurine appears to exert a significant effect on energy expenditure, by decreasing the expression of PGC-1α in the white adipose tissue (WAT) and by increasing its expression in brown adipose tissue (BAT), this way favoring thermogenesis (101). Focusing on the taurine-mediated regulation of transcription factors, it has been proven that taurine supplementation causes an increase in the transcriptional activation of PPARα and UCP2, thereby preventing WAT formation and promoting BAT formation in high-fat/cholesterol-fed mice (102). Therefore, taurine prevents fat hyperplasia through its regulatory impact on PGC-1α, an important source of energy expenditure in adipose tissues (102). In addition, it has been shown that taurine supplementation prevents adipocyte differentiation (103) and modulates the expression of adipokines by inhibiting the signal transducer and activator of transcription STAT3 signaling pathway (104). Notably, taurine modulates the expression levels of adipokines through its conversion to taurine chloramine (104). In addition, it should be noted that the duration of taurine treatment appears to be a major determinant for the control of obesity, in the sense that prolonged administration of taurine has produced more stable and prominent results than taurine's temporary treatment for 6 weeks (105).

Treating hyperlipidemia is one of the main targets in the management of type 2 diabetes. In stroke-prone spontaneously hypertensive rats (SHRSP) on a high-cholesterol diet (106), mice on a high-cholesterol diet (107), rats on a high-cholesterol diet (108) and ovariectomized rats (109), the administration of taurine was shown to exert both a hypolipidemic and a hypocholesterolemic effect, via three distinct mechanisms. The first mechanism of taurine seems to be based on a reduction in bile acid absorption from the intestine (110), by increasing the cholesterol 7 alpha-hydroxylase (CYP7A1) bile acid synthesis enzyme and by stimulating the fecal release of bile, in rats fed a high-cholesterol diet (106). The second mechanism includes an increase in the binding of LDL to the LDL receptor from blood (111), and the third mechanism has been shown to involve the inhibition of very-low-density lipoprotein (VLDL) secretion from the liver (112), accompanied by the inhibition of hepatic acyl-CoA:cholesterol acyltransferase (ACAT) activity (111). In addition, taurine appears to exert its hypolipidemic effect through the upregulation of hepatic LDL receptors and by ensuring accelerated LDL turnover (113) and a reduction in serum leptin levels (70). Another hypolipidemic mechanism has been associated with a reduction in triglycerides; as the administration of taurine has been shown to enhance the activity of LPL in both plasma and liver, thereby promoting the peripheral clearance of triglycerides (114). Indeed, taurine supplementation has been shown to enhance peroxisomal fatty acid β-oxidation and to reduce fatty acid synthase activity in the livers of type 2 diabetic/obese mice, contributing to normal redox homeostasis with low MDA formation (115). As a result, the hypolipidemic effect of taurine has been attributed to its ability to activate mitochondrial fatty acid oxidation.

Hence, the hypolipidemic effect of taurine is accompanied by a marked decline in leptin levels and by bypassing insulin resistance in type 2 diabetic rats (70). In OLETF rats with type 2 diabetes, taurine has been noted to ameliorate hyperglycemia and dyslipidemia, by enhancing insulin sensitivity and by inhibiting leptin secretion (70). In the same frame, chronic taurine administration (9 weeks) to 2-month-old OLETF rats has been shown to lead to the elimination of serum triglycerides and cholesterol without altering LPO (116). This makes sense if one considers that taurine has been shown to positively modulate hypothalamic neuropeptide expression (117) and to normalize the 24-h pattern of leptin production via the regulation of insulin levels (118). Therefore, it has been revealed that taurine functions in the hypothalamus of OLETF rats by suppressing food intake and locomotor activity, and by stimulating signal transduction through the protein kinase B/Forkhead box (Akt/FOX) (117). Another study by Carneiro *et al* (48) demonstrated that taurine regulated glucose homeostasis through the control of leptin gene expression levels, which are required for glucose-stimulated insulin secretion.

### Protective effect of taurine in animal models subjected to glucose or fatty acid infusion

The protective effects of taurine against diabetic stress-mediated pancreatic islet dysfunction have been evaluated in animal models subjected to glucose or fatty acid infusion. Both *in vivo* and *in vitro* experiments have demonstrated that taurine inhibits oxidative stress, improves pancreatic β-cell dysfunction, thereby conferring enhanced sensitivity to insulin in rats following lipid infusion (48 h of oleate infusion) (80). Other studies have supported that taurine stimulates Akt in hepatic cells, causing alterations that lead to restoring the function of pancreatic β-cells and to ameliorating insulin resistance in mice fed a high-fat diet (HFD) (102,119). Particular emphasis has been given on the ability of taurine to enhance insulin sensitivity by negatively affecting hyperglycemia through the activation of the PI3K/Akt signal transduction pathway (102,119,120). In a previous study, a 48-h combination treatment scheme, including both oleate and taurine was shown to restore insulin release from pancreatic islets, mainly due to the ROS-scavenging capacity of taurine (80). Additional experiments have proven that taurine contributes to the maintenance of insulin signaling by interfering with fatty acid-induced oxidative stress, JNK1 activation in pancreatic islets and livers subjected to intravenous infusion of fatty acids (91).

Furthermore, several studies have supported the significance of taurine as a promising agent in controlling oxidative stress in diabetic conditions; however, the vast majority of studies have only evaluated its efficacy in animal models and clinical trials in the very narrow window of up to 6 months (73,80,121). The long-term administration of taurine has a distinct differential effect on glucose and lipid levels in diabetic conditions. For example, Branco *et al* (122) demonstrated that taurine supplementation over 12 months caused adverse effects in lipid and glucose tolerance in mice fed a HFD. Similarly, long-term 5% taurine supplementation caused ectopic lipid accumulation, hyperglycemia, pancreatic islet hyperfunction, insulin resistance and signs of renal damage. Renal injury in HFD-fed mice that received taurine was identified by increased rates of urinary proteins and albumin (123). However, the persistently high concentration of taurine interfered with glomerular and tubular processes, negatively affecting glomerular filtration barrier integrity, causing the development of vacuoles in renal tubules, ultimately resulting in disturbed renal reabsorption and renal dysfunction (124). The development of vacuolar structures were shown to be involved in the storage of phospholipids and cholesteryl esters in proximal tubular cells (124). By contrast, short-term taurine supplementation appeared to decrease renal damage and to prevent the increase of blood urea nitrogen (BUN) and serum creatinine levels in diabetic rats (125). Consequently, taurine-based therapies for obese and diabetic subjects should be carefully designed to confer the desired therapeutic effect, which aims at the impaired glucose control due to prolonged taurine supplementation.

The beneficial effect of taurine against diabetic stress has also emerged in various combination schemes. For example, dietary fish oil containing n-3 PUFAs (eicosapentaenoic acid and docosahexaenoic acids) in combination with 4% taurine dietary supplementation proved to be very successful against white adipose tissue formation and high blood glucose levels in KK-Ay mice (115). Characteristically, the effect caused by the combination scheme was significantly more prominent than that mediated by soybean oil treatment alone (115). In another study where 2% taurine was administered in combination with fish oil to obese mice, it was shown that fat metabolism was mediated by fatty acid oxidation and glucose uptake (115,126). Accordingly, Kishida *et al* (109) proved that taurine was capable of decreasing plasma total cholesterol concentration in rats that were fed with corn oil, but not in rats fed with coconut oil. Taurine appeared to be efficient in maintaining normal liver lipid levels by enhancing the activity of LDL receptor (LDL-R) in the liver (109).

## 4. Effect of taurine on the fructose-fed rat model

Dyslipidemia, insulin resistance and type 2 diabetes can be simulated with the administration of a high-fructose diet to rodents (127), as also indicated by studies on humans (128). A high-fructose diet in rats has been shown to cause a marked decline in plasma and liver taurine levels (114). In this context, previous studies have highlighted the important contribution of taurine to bypass fructose-mediated insulin resistance (129,130).

Taurine supplementation has been proven to be highly efficient in controlling glucose production in fructose-fed rats, independently of treatment duration frames (30 or 182 days). On the one hand, improved glucose intolerance and insulin resistance were observed in Wistar rats following long-term (26 weeks) fructose and 2% taurine supplementation (131). Despite being able to restore triglycerides to normal cells in fructose-fed rats, taurine supplementation did not seem to alter hepatic phosphoenolpyruvate carboxykinase (PCK1) mRNA levels (131). No effect was also observed on phosphorylated Akt levels in the skeletal muscle of fructose-fed rats, which was in accordance with the observation that taurine acts as an agonist of insulin signaling (74). In the same frame, there was no recorded activation of the rate-determining gluconeogenic enzyme PCK1(132), thereby excluding the possibility that increased glycogenolysis is the main factor for the increase in glucose. Those findings were not in agreement with previous results that supported the inhibitory action of taurine on glycogenolysis (133), even though such discrepancies could be due to differences in animal strains, and doses and duration of taurine treatment and so on. Therefore, the improvement in glucose tolerance in taurine/fructose-fed rats was attributed to the increased release rates of insulin (48). Taurine has also been shown to confer insulin sensitivity, by reducing lipid peroxidation in fructose-fed rats (129,134). In accordance with this, taurine supplementation to high fructose-fed rats has been shown to prevent the formation of lipid peroxidation products, to enhance insulin sensitivity and to suppress the accumulation of glycated proteins, such as fructosamine and HbA1c, which are localized in the plasma of these animals (115,129,134).

## 5. Therapeutic effect of taurine on diabetes

### Clinical effectiveness of taurine on type 1 diabetes

From a historical point of view, Franconi *et al* (67) were the first to provide insight into the significance of taurine against type 1 diabetes, based on the observation that taurine expression levels were very low in the plasma of diabetic patients. Specifically, patients with type 1 diabetes (n=39) who were administered taurine at doses of 1.5 grammars per day for 90 days (long-term treatment) did not exhibit any alterations in their glucose metabolism (67). The ability of taurine to reduce platelet aggregation in diabetic patients was also highlighted, as shown by the dose-dependent restoration of taurine concentration in the plasma and platelets of type 1 diabetes subjects (67). Following this, Elizarova and Nedosugova evaluated the effects of taurine on a small subset of patients with type 1 diabetes (n=10), who had already been treated with insulin (13); in this case, taurine was administered in doses of 0.5 g per day for 30 days and was shown to improve the symptoms of type 1 diabetes, as demonstrated by an increase in carbohydrate metabolism and a decline in triglyceride content (13). Another clinical study demonstrated the therapeutic effectiveness of taurine against insulin resistance due to its ability to reduce oxidative stress (73). Specifically, a 2-week taurine supplementation of 3 g per day for 48 h before lipid-infusion was shown to prevent the formation of LPO products and to improve pancreatic β-cell function (73).

It is well-established that the intact vascular endothelium produces nitric oxide (NO), which is regarded as a vasculoprotective molecule, by inhibiting platelet, as well as leukocyte adhesion to the vascular endothelium (135). In diabetic conditions, hyperglycemia is responsible for suppressing the endothelial NO synthase (NOS), contributing to excessive ROS formation; however, it also enhances the overproduction of vasoconstrictor substances, such as endothelin 1 and the activation of the renin-angiotensin system (136). In this context, a cross-over study employing male patients with type 1 diabetes who were administered taurine at doses of 1.5 g per day, revealed that the particular treatment scheme was able to reverse early, detectable conduit vessel abnormalities in the endothelium of these patients (137); in support of this, a study using diabetic animal models suggested a possible role for taurine on suppressing monocyte-endothelium interaction (138). In another study, the co-culture of human umbilical vein endothelial cells (HUVECs) with monocytes isolated from smokers revealed signs of impaired monocyte-endothelium interaction (139). Taurine administration appears to restore this interaction by decreasing the efflux of NO from the monocytes of smokers, leading to elevated endothelin-1 in HUVECs (139). In a similar context, the hypoglycemic effect observed in patients with type 1 diabetes who received taurine, has been attributed to improved endothelial function (137). Specifically, taurine supplementation at doses of 1.5 g per day for 2 weeks was able to reverse all the alterations related to arterial stiffness in diabetic patients (137). Notably, the same group had previously reported that this particular mode of taurine supplementation (1.5 g per day for 2 weeks) has been shown to improve the disturbed flow-mediated dilatation flow of the brachial artery in young cigarette smokers (139).

### Clinical effectiveness of taurine on type 2 diabetes

Taurine has been shown to exert a therapeutic effect on obesity and lipid profile in clinical trials. For example, the administration of taurine at doses of 3 g per day for 7 weeks has been shown to improve plasma triglyceride and total cholesterol content in overweight non-diabetic subjects (140). In another case, the administration of taurine at doses of 6 g per day in subjects on high-fat and high-cholesterol diets appeared to improve the symptoms of diabetic complications (141). Furthermore, other trials have reported that taurine supplementation at doses of 3 g per day for 4 months does not have any effect on glucose and lipid peroxide levels on patients with type 2 diabetes (72). However, the HbA1C appears unaltered in diabetic patients following the taurine supplementation of 1.5 g per day for 8 weeks, therefore preventing pancreatic β-cells from being more sensitive to insulin (72); these findings are not in agreement with the results of certain animal studies. In terms of diabetic nephropathy, Nakamura *et al* (142) reported that the long-term supplementation of taurine, (3 g per day) did not have any effect on the phenotype of patients with microalbuminuria related to type 2 diabetes within 12 months, as demonstrated by the relative expression levels of fibrotic markers [serum collagen IV and plasma matrix metalloproteinase-9 (MMP)-9]. Notably, the accuracy and validity of the results obtained in clinical studies are questioned by the presence of certain limitations, such as the concomitant administration of other medications, the severity of the disease, the correct patient characterization and stratification, the taurine dosage scheme, and the duration of trials among others (59).

## 6. Functional significance of taurine in renal disorders

Initial studies on taurine using ion-exchange chromatography in 1960 revealed that a significant amount was localized in the urine of patients with renal tubular disorders or with hereditary aminoaciduria (143). Following this, the increased excretion of taurine was also detected in patients with X-linked disorders and genetic syndromes such as Fanconi anemia and cystinosis (143).

Taurine is a key modulator of several physiologic functions in renal cells. It has been shown to positively affect ion reabsorption and secretion, urine composition, renal blood flow, osmoregulation and glomerular filtration (144). In renal cells, taurine has been shown to exert major non-ionic osmolarity capacity. Several lines of evidence have pointed out that taurine, along with betaine, myoinositol and a-glycerolphosphate, enable the normal flux of the renal medullary tonicity gradient (145). In this context, it has been hypothesized that taurine may represent an important therapeutic agent in cases of renal dysfunction.

## 7. Effects of taurine on hypertension

Hypertension is a prevalent symptom in renal disorders, and it is considered to occur through defects in the renin-angiotensin-aldosterone system (RAAS), monogenic abnormalities of ion transporters and acute kidney inflammation (146).

As taurine is well-known for its antioxidant properties, its potent osmoregulatory potential and its regulatory importance for cation transport in renal cells (147), it has also been extensively investigated in hypertension. Taurine supplementation seems to reverse the adverse effects of hypertension in animal models (71,121,148-151) and importantly, the onset of hypertension in rats has been accelerated in conditions of taurine loss (152). In this direction, it has been demonstrated that taurine exerts an inhibitory effect on adriamycin-mediated proteinuria and hyperlipidemia, by preventing urinary taurine excretion (153). Other researchers have reported that taurine negatively affects lipid levels, reducing the efficacy of enzymes [lecithin cholesterol acyltransferase (LCAT), LPL] or serum factors [platelet-activating factor (PAF)] or GSH values, in an attempt to counterbalance renal dysfunction (154). In a previous study, in a fawn-hooded hypertensive rat model of spontaneous hypertension, taurine supplementation appeared to suppress proteinuria, through its capacity to increase urinary NO excretion, as well as sodium (Na^+^) and potassium (K^+^) excretion (155).

An important mechanism through which taurine exerts its hypotensive effect is through the RAAS, which is classified as the major reason for the development of hypertension (156). The hypotensive ability of taurine mainly relies on antagonizing angiotensin II, thereby prohibiting the activation of the RAAS, as demonstrated in both cell cultures (157,158) and animal models, such as i) spontaneously hypertensive rats (SHR) (159,160); ii) rats fed a high-fructose diet (129); and iii) deoxycorticosterone acetate (DOCA)-salt hypertensive rats (161). On a similar note, taurine has been shown to be effective against lead-induced hypertension (162) and cyclosporine A-induced hypertension (148).

The anti-oxidant and anti-inflammatory properties of taurine also appear to protect renal cells in models of hypertension. In the case of *N*-nitro-L-arginine methyl ester in Sprague-Dawley rats, taurine appears to be an attractive agent against the form of hypertension, by reducing pro-inflammatory cytokine expression levels, whilst promoting NOS activity, thereby positively regulating serum NO levels (151).

Taurine supplementation has been shown to attenuate the advancement of chronic kidney disease (CKD), most probably due to positively affecting renal blood flow dynamics, as well as reducing hypertension and proteinuria. While focusing on the molecular mechanisms underlying the beneficial effect of taurine against hypertension, it has been proposed that taurine reduces vascular resistance and positively controlling arterial blood pressure through regulation of the autonomic nervous system (151,163). Τaurine has been reported to be involved in the regulation of the sympathetic nervous system, where it has been shown to lead to a marked decline in hypertension in both rats and humans (164,165). Considering that the RAAS and renal sympathetic nerve activities participate in the renal excretion of fluids and sodium (166), it can be hypothesized that the diuretic and natriuretic properties of taurine are the direct results of the suppression of either the RAAS or the renal sympathetic nerve activity.

Taurine appears to negatively affect hypertension through its action on the hydrogen sulfide (H_2_S) content. Of note, increased taurine levels have been closely associated with increased H_2_S levels, thereby leading to hypotension manifested by reduced transient receptor potential channel 3 (TRPC3)-induced signaling in the vasculature (167).

Taurine has been shown to improve vessel function and endothelial function. The hypotensive properties of taurine have been observed in both animal models and in hypertensive human subjects, where taurine reduces blood pressure through its effects on endothelial cells (167,168). Following taurine supplementation, improved endothelial function and reduced oxidative stress have been observed (167,168). For example, the taurine-mediated attenuating effect on endothelial-dependent vasodilation was documented in young smokers following taurine supplementation (1.5 g) for 5 days (139) and in rats following treatment with 1% taurine for 8 weeks (169). The underlying mechanism of taurine's action was based on reducing the calcium overload, attenuating oxidative stress and reducing sympathetic/inflammatory action, resulting in improved kidney function (71,147,148,151,152,161,168,170,171). Consistent with the above, it has been shown that taurine can possess diuretic and natriuretic properties in saline-loaded rats (152).

In a clinical setting, Ogawa *et al* (172) supported the view that patients with hypertension are characterized by low plasma levels of taurine. Accordingly, an epidemiological study indicated that there was an inverse association between taurine intake and blood pressure (173). The administration of taurine (6 g) in young patients with borderline hypertension led to a reduction in both systolic and diastolic blood pressure within 7 days (121). The same favorable effect was reported when taurine (3 g) was administered for a longer period (2 months) (174). Recently, Sun *et al* (167) demonstrated a marked decline in diastolic/systolic pressure in 120 pre-hypertensive subjects following taurine supplementation.; in particular, high diastolic pressure (80-89 mmHg) was reduced to 4.7 mmHg following taurine administration (1.6 g/day) to a pre-hypertensive subject for 12 weeks (167). In the same patient, systolic pressure ranged from 120 to 139 mmHg prior to taurine administration, whereas it appeared to decrease to 7.2 mmHg following its administration (167). Moreover, taurine supplemented (6 g/3 weeks) to healthy volunteers has been shown to be inversely associated with levels of urinary norepinephrine excretion (141)

## 8. Effects of taurine on acute kidney injury

Acute kidney injury (AKI) is a very severe health issue in humans, constituting a major cause of mortality (175). A significant number of studies have highlighted the potential of taurine to be used as a novel therapeutic agent, as well as a significant diagnostic factor in renal injury.

Several nephrotoxins can lead to the development of AKI and include heavy metals, such as lead, cadmium, mercury, uranium and gold. In this respect, taurine has been shown to eradicate renal toxin-induced symptoms, thereby acting as a potent reno-protective agent (40). For example, in a subchronic lead intoxication rat model, exposure to lead caused the animals to be more susceptible to brain oxidative stress, as shown by reduced delta-aminolevulinic acid dehydratase (ALAD) activity, diminished GSH values and upregulated zinc protoporphyrin (176); taurine appeared to salvage rats from the harmful injuries of lead exposure via normalization of the GSH status (176). In another study investigating the penetration rates of trace elements (Se, Cu, Fe^2+^, and Mn) using ICP-MS in arsenic-exposed rats, the administration of taurine appeared to reverse the toxic changes in the kidneys and livers of these animals (177). This makes sense if one considers that taurine is composed of an amino group and a sulfonate group instead of a carboxyl group, thereby promoting the excretion of heavy metals and their conjugation to other compounds (178), as well as arsenic excretion.

Among toxins, arsenic is an established factor in causing acute kidney injury due to mitogen-activated protein kinase (MAPK)/NF-kB activation and mitochondrial-dependent pathway alterations (179). Even in low concentration doses, arsenic is able to cause an oxidative burst and to enhance LPO and protein carboxylation in renal cells (179), as well as to cause oxidative DNA damage to vascular smooth muscle cells (180). Taurine has been shown to confer significant kidney protection from oxidative DNA damage in arsenic-intoxicated mice (181). Following the administration of taurine to mice, renal tissues were characterized by low 8-hydroxy-2-deoxyguanosine (8-OHdG) in their glomeruli and renal tubule areas (181). The weak distribution of 8-OHdG immunoreactivity was in agreement with the histopathological changes observed in arsenic-exposed mice that received taurine, as demonstrated by the lack of DNA strand breaks in the renal tissues in immunohistochemistry data (181). Consistent with the above, in another study, taurine appeared to reverse intestinal DNA damage in rats following potassium bromate exposure (182) and to prevent testicular oxidative injury in diabetic rats, by mitigating LPO and DNA damage levels (62).

ΑKI can also be caused experimentally by the administration of acetaminophen (183). Acetaminophen-administered swiss albino mouse strains are usually characterized by renal necrosis and significant alterations in normal oxidative status (183). Treatment of these animals with taurine has been shown to improve the signs of nephrotoxicity, as evidenced by a reduction in CYP2E1 expression (183).

In cases of antibiotic-mediated nephrotoxicity, evidence of kidney injury includes elevated BUN and urinary *N*-acetyl-glucosamine (NAG) and reduced Na^+^ K^+^ ATPase activity (184). For example, the co-administration of taurine and quercetin has been shown to normalize creatinine clearance and to decrease protein urinary excretion, uronic acids, and urinary NAG in animal models induced by the combination of gentamicin and diclofenac (184). Cortical LPO products appear to increase after administration of gentamicin and to decline after the administration of taurine and quercetin (184).

In radiation-induced kidney injury, taurine and its transporter can be restored to normal levels in renal cells following taurine supplementation (185). In glomerular disease, taurine is converted to taurine chloramine, via the increased intracellular activity of myeloperoxidase in invaded polymorphonuclear leukocytes (186). Taurine chloramine has been shown to function as an oxidant reservoir, exhibiting antioxidant effects in both proximal or distant sites (186). This response is more prominent in phagocytes, which contain a high number of taurine-related anti-oxidants (187), particularly during the early stages of inflammation in the glomeruli and tubules of the renal tissues (188).

## 9. Effects of taurine on diabetic nephropathy

Human diabetic nephropathy is a severe health concern that often requires renal dialysis (189). End-stage renal disease requiring dialysis is most often caused by diabetic nephropathy. A number of different mechanisms have been identified as essential components to the development and progression of diabetic nephropathy. Hyperglycemia is considered to be the common underlying driving force for diabetic nephropathy through the following mechanisms: The overexpression of glucose transporters, glucose accumulation in mesangial cells and ECM production (189). In this manner, signals are transmitted in specific transduction pathways that lead to an overproduction of ROS, pro-inflammatory cytokines and hormones, thereby conferring pro-inflammatory characteristics to patients with diabetes (190). The aberrant glomerular and tubular structural changes are observed in diabetic nephropathy. In particular, glomerular basement membrane thickening, glomerulosclerosis and tubulointerstitial fibrosis seem to occur due to the accumulation of AGEs in the kidneys (191). Furthermore, persistent hyperglycemia accounts for the activation of PKC, which in turn leads to both transforming growth factor β1 (TGF-β1)-mediated-ECM production in mesangial cells and increased eicosanoid release linked to glomerular hyperfiltration (192). Notably, moderate hyperglycemia without glycosuria can enhance plasma renin activity and mean glomerular pressure, resulting in hyperfiltration and glomerulosclerosis (189).

**Figure 3:**
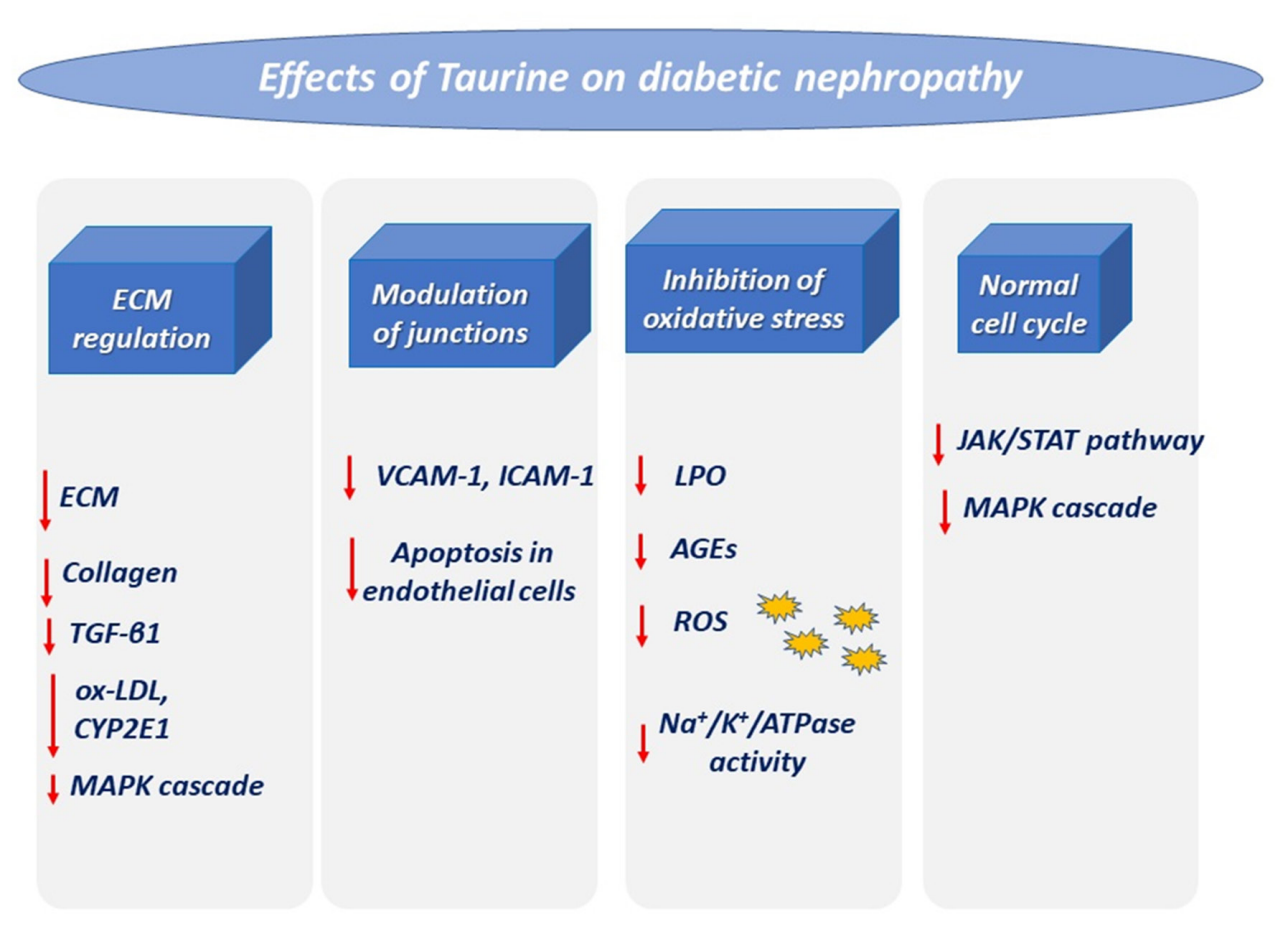
Effects of taurine on diabetic nephropathy. Diabetes triggers impaired kidney function via the mentioned pathways, which are downregulated via treatment with taurine. ECM, extracellular matrix; TGF-β1, transforming growth factor β1; ox-LDL, oxidized low-density lipoprotein; CYP2E1, cytochrome P450 2E1; MAPK, mitogen-activated protein kinase; VCAM-1, vascular cell adhesion molecule-1; ICAM-1, intercellular adhesion molecule-1; LPO, lipid peroxidation; AGEs, advanced glycation end-products; ROS, reactive oxygen species.

Taurine has been shown to exhibit great potential in inhibiting the progression of diabetic nephropathy (19), given that taurine deficiency is considered a key characteristic in diabetic patients (69). Of all renal disorders, taurine has been studied most extensively in diabetic nephropathy as a possible new therapeutic strategy (summarized in Fig. 3). This has been accomplished in several diabetic animal models induced by STZ or alloxan, where the administration of taurine has caused a regression of diabetic symptoms by inducing hypoglycemic, anti-oxidant and renoprotective activity, including improved hyperglycemia, dyslipidemia, as well as reduced blood HbA1c 1c levels and oxidative stress (24). It should be highlighted that the beneficial effect of taurine in STZ-animal models is crucial (33), given that the particular animal model can mimic all the complications of human diabetic nephropathy (193).

The major mechanism underlying the cytoprotective role of taurine against diabetic nephropathy is the activation of antioxidant enzymes. Of note, the protein expression levels of HO-1 appear to be elevated in the renal glomeruli of diabetic rats (194). The administration of taurine alone or in combination with other anti-oxidants is able to relieve common symptoms of diabetes, mainly by normalizing HO-1 expression (194). In this manner, taurine can salvage renal glomerular cells from pathological changes and inhibit renal disturbances such as proteinuria and hypertension (194). Furthermore, the nephroprotective property of taurine can be attributed to its ability to decrease renal nicotinamide adenine dinucleotide phosphate (NADPH) oxidase activity (22) and to counteract the upregulation of plasminogen activator inhibitor-1(195).

The beneficial effect of taurine on diabetic renal symptoms may also occur via the modulation of various signal transduction pathways. For example, in high glucose conditions, hypertrophic renal tubular epithelial cells seem to revert to their normal size following administration of taurine, which inhibits JAK2, signal transducers and STAT1/STAT3), as well as extracellular-regulated kinase (ERK)1/2 kinases (196). Taurine has been shown to inhibit fibronectin and type IV collagen synthesis and expression of cyclin D/cdk4 and to reduce p21 Waf1/Cip1 and p27 (Kip1) levels (196). In this manner, taurine has been shown to slow down the hypertrophy of high-glucose-treated renal cells, and to induce their proliferation through alterations in signal transduction pathways and the ECM (196). Hence, taurine improves the protein values for reactive AGEs in renal cells grown in the high-glucose culture medium, due to its anti-fibrotic action, which is mediated by the inactivation of Raf-1/ERK (197).

Furthermore, taurine has been shown to be a significant renoprotective agent in rats with alloxan-induced diabetes due to its potent antioxidant and anti-inflammatory properties. In particular, it protects renal cells from apoptosis and inflammation, by significantly reducing the expression levels of pro-inflammatory cytokines [TNF-α, interleukin (IL)-6 and IL-1β] and by decreasing NADPH levels (22).

Importantly, taurine mitigates diabetic complications by interfering with leukocyte adhesion molecules (33). In STZ-treated diabetic rats characterized by renal injury through increased levels of BUN, serum creatinine and renal MDA, taurine has been shown to reverse all the histological changes through the downregulation of LOX-1 and ICAM-1(33). In rats with STZ-induced diabetes, following 4 months of established diabetic nephropathy, the administration of taurine (1%) appeared to improve diabetic complications, as evidenced by diminished proteinuria. This was accompanied by a marked decline in TGF-β1 expression in the renal glomeruli of diabetic rats, improved mesangial ECM expansion, and a further increase in protein urinary excretion (198). Accordingly, LPO and TGF-β1 values also seem to decrease following the administration of taurine in renal proximal tubule cells grown under high glucose conditions (199). Additional mechanisms accounting for the beneficial effects of taurine on high-glucose-cultured renal cells include changes in the MAPK signal transduction cascade and STAT3 transcription factor (196). Such data provide sufficient evidence to support the immense potential of taurine to be used as an effective therapeutic agent in patients with established diabetic nephropathy. Indeed, it has already been demonstrated to protect renal tubular epithelial cells of STZ-treated diabetic rats or renal tubular cells from hypertrophy and fibrosis (200). It has also been proposed that renal tubular cell hypertrophy may be caused by the accumulation of AGEs via reduced NO/cyclic guanosine monophosphate/cGMP-dependent protein kinase (NO/cGMP/PKG) signaling. Even in this case, taurine also seems to reverse hypotrophy, mediating the activation of NO/cGMP/PKG signaling (200). Nephropathy is improved via CYP2E1 expression and activity in diabetic rat kidneys, as CYP2E1 activity is capable of creating an oxidative environment and inducing the metabolism of various endogenous and exogenous compounds, ultimately resulting in formation of AGE products and ROS accumulation (201).

Apart from the protective action of taurine alone, it has been shown that a metformin and taurine combination scheme enhances insulin sensitivity, causing a marked decline in the high glucose levels observed in diabetic patients (80). The underlying mechanisms of action probably involve the activation of ECM degradation and the attenuation of vascular oxidative stress. The combination scheme has proven to be efficient in inhibiting the renin-angiotensin system that is mediated by hyperglycemia and in releasing smaller amounts of TGF-β1, which is ultimately involved the development of interstitial fibrosis and mesangial and tubular hypertrophy (202). These findings are in agreement with the findings of experiments where taurine has been used alone and not in combination; i.e., effective in reducing the TGF-β1 mRNA expression in rats with experimental non-alcoholic steatohepatitis (203) and in rats with carbon tetrachloride (CCL_4_)-induced hepatic fibrosis (204). This protection was also attained with the metformin/taurine combination treatment scheme, through a reduction in MDA formation and an improvement of the GSH/GSSG ratio in the plasma and liver of diabetic rats (202). Indeed, it has been documented that taurine administration reduces gluconeogenesis and contributes to normal homeostasis, through the normalization of GSH levels and a reduction in hydroxyl free radicals (202).

Despite reported advancements being made in the field of therapeutics for diabetes, novel approaches are required for a more accurate diagnosis and the prevention of renal injury. Understanding more precisely the underlying molecular mechanisms of the pathogenesis of diabetic nephropathy is a prerequisite for devising preventive strategies and in enhancing the therapeutic efficacy of existing drugs. Some progress has been made in animal modeling, strain analyses, genotype associations and pathologic processes (205); however, the ideal model of diabetes has yet not been created.

## 10. Effects of taurine on renal transplantation

Renal allografts currently constitute the main option for the restoration of normal renal function. A previous study including 11 children who had received kidney transplants, demonstrated that plasma taurine and leucine concentrations were lower in those children at 97±14 days post-transplantation surgery, as compared to 10 age-matched controls. Several muscle amino acids, including taurine, were increased post-transplantation. The authors postulated that classical used glucocorticoid (prednisone) may have affected the plasma taurine status (206). In this manner, donor preconditioning with taurine has been shown to rescue kidney grafts from the harmful consequences associated with transplantation procedures (206).

A significant challenge in transplantation is the composition of the perfusion solution that is injected in cadaveric kidneys prior to the allograft procedure. Initially, the addition of taurine to the perfusion solution seems to significantly improve hypoxic changes, to restore reoxygenation and to allow the recovery of energy metabolism in LLC-PK1 cells (207). Those properties were also reinforced by the regulatory effects of taurine on calcium levels, thereby suggesting that taurine can enhance the cellular growth of transplants (207). Nonetheless, ischemia/reperfusion constitutes a significant issue during renal transplantation, as blood vessel clamping seems to activate antioxidant injury to the renal vasculature, including the endothelium. For example, in a previous study, in rats subjected to ischemia for 60 min, followed by 90 min of reperfusion, energy biosynthesis seems to slow down, as evidenced by increased creatinine and decreased ATP values; in this case, the taurine administration of 40 mg/kg was reported to decrease only creatinine, and not ATP levels (208). In support of the beneficial effects of taurine on ischemia/reperfusion, another study demonstrated that taurine improved endothelial damage, as indicated by the increased formation of ROS, the disrupted calcium regulation and reduced endothelial NOS activity. Accordingly, taurine has been demonstrated to improve existing histopathological renal injury abnormalities, by increasing the BUN and creatinine content in Wistar rats (209). In these animals, treatment with taurine was shown to protect kidney transplants from apoptotic cell death and triggers anti-oxidant responses. Following transplantation, the taurine-treated tissue was characterized by decreased caspase-3 levels as compared to the controls, while SOD and heat shock protein levels were increased. The serum levels of creatinine, aspartate aminotransferase (AST) and lactate dehydrogenase (LDH) appeared to be markedly reduced in a dose-dependent manner following the treatment of kidney transplants with taurine (210). Moreover, the use of the calcineurin inhibitors cyclosporine (CsA) and tacrolimus (FK506) seems to be essential in eliminating the danger of organ rejection. Despite their reported necessity, the use of calcineurin inhibitors is hindered by ROS accumulation, which may ultimately lead to the induction of hypertension and nephrotoxicity (148). This damaging action may be prevented with taurine, as *in vivo* experiments in rats have demonstrated the restoration of antioxidant responses (GSH, GPx and SOD), as well as normal serum creatinine and proteinuria levels following taurine supplementation, thereby contributing to normal renal transplant functioning. In this case, the underlying mechanism in counteracting CsA-mediated cytotoxicity was postulated to be the ROS-scavenging activity of taurine.

## 11. Conclusions

In diabetes, the beneficial effects of taurine have been reported in several animal models and multiple mechanisms seem to be involved. In type 1 diabetes, taurine exerts its beneficial effects through its antioxidant, anti-inflammatory effects and its effect on increasing glutathione levels, augmenting insulin secretion, activating inhibitory neurotrasmitters and interfering hypothalamic-pituitary-gonadal axis.

In type 2 diabetes, taurine can act either as a hypoglycemic or an anti-obesity or a hypo-lipidemic agent. The hypoglycemic effect of taurine is manifested through its antioxidant, anti-inflammatory and insulin-sensitizing properties. The anti-obesity effect is manifested via increasing the oxygen consumption rate, whereas the hypo-lipidemic effect relies on promoting cholesterol degradation or increasing LDL uptake from blood and on lowering cholesterol release from the liver or reducing bile acid absorption from the intestine. Even though the beneficial effects of taurine on glucose and lipid metabolism have been reported, there is an inconsistency in the results of various studies, mainly due to the concentration of taurine used in each experimental set-up. Some other differences are focused on the different animal strains, variations in the supplementation, the duration of treatment, different handling and living conditions of animal models, the different types of dietary fatty acids and other random events during experimental procedures. Epidemiological studies have indicated the potential therapeutic usefulness of taurine in ameliorating diabetic 1 and 2 complications in humans. Nevertheless, the clinical trials conducted to date are limited, and there is an insufficient amount of data on the correct dosage of administration, the duration of trials, sample size, the severity of the disease, the presence of metabolic syndrome-related diseases, the basal level of taurine intake from food, lifestyle factors and genetic background. Additional studies need to be designed with optimized clinical trial conditions, in order to overcome the aforementioned hurdles. This will provide important information as to whether taurine can be recommended unequivocally as a nutraceutical for the prevention of diabetes.

Taurine exerts a cytoprotective and multifaceted effect on the homeostasis of renal cells due to its antioxidant and its osmoregulatory properties. Indeed, the protective nature of taurine is proved to be invaluable in animal models of renal disorders, mainly through its antioxidant and osmoregulatory properties. For example, the protective effect of taurine against hypertension, which is commonly observed in renal disorders, is substantiated through its inhibitory action on the RAAS and in the sympathetic nerve system, as well as by its antioxidant, its anti-inflammatory and its ameliorative effect on endothelial dysfunction. Furthermore, the beneficial role of taurine against diabetic nephropathy is advocated through its interference with the MAPK pathways, and its antioxidant and its anti-inflammatory nature. The present review article highlights the importance of taurine in circumventing side effects in patients with kidney transplantation. Even though taurine has been employed in multiple nephrotoxic animal models, its therapeutic use is limited, enabling for further research regarding the molecular mechanisms underlying the ameliorative nature against signs of renal injury.

## Data Availability

Not applicable.
